# Direct Production of Difructose Anhydride IV from Sucrose by Co-fermentation of Recombinant Yeasts

**DOI:** 10.1038/s41598-019-52373-5

**Published:** 2019-11-04

**Authors:** Hyunjun Ko, Jung-Hoon Bae, Bong Hyun Sung, Mi-Jin Kim, Soon-Ho Park, Jung-Hoon Sohn

**Affiliations:** 1 Synthetic Biology and Bioengineering Research Center, Korea Research Institute of Bioscience and Biotechnology (KRIBB), 125 Gwahak-ro, Yuseong-gu, Daejeon 34141 Republic of Korea; 2 Department of Biosystems and Bioengineering, KRIBB School of Biotechnology, Korea University of Science and Technology (UST), 217 Gajeong-ro, Yuseong-gu, Daejeon 34113 Republic of Korea

**Keywords:** Biopolymers, Expression systems

## Abstract

A functional sweetener, difructose anhydride IV (DFA IV), is enzymatically produced from sucrose via levan by levansucrase (LSRase) followed by levan fructotransferase (LFTase). Here, we have demonstrated a consolidated production system for the direct conversion of DFA IV from sucrose using the co-culture of two recombinant yeast strains secreting LSRase from *Bacillus subtilis* and LFTase from *Arthrobacter ureafaciens*, respectively. To ensure secretory production of the enzymes, target protein-specific translational fusion partners (TFP) were employed, and the selected strains produced 3.8 U/mL of LSRase and 16.0 U/mL LFTase activity into the fermentation broth. To optimise the direct production, sucrose concentration and cell ratios were investigated. In the optimised conditions, 64.3 g/L crude DFA IV was directly produced from 244.7 g/L sucrose using co-fermentation of recombinant yeasts. These results promise an efficient production titre, yield, and DFA IV productivity in an industrially applicable method.

## Introduction

Given the increased interest in functional foods, low-calorie and functional sweeteners have become more popular than sucrose. Minimal cyclic fructose dimers, such as difructose anhydrides (DFAs), have been applied as functional sweeteners owing to their promising prebiotic effects, good antioxidant and immunoregulatory effects, and increased absorption of calcium in vertebrates with half the sweetness of sucrose^1–3^. There are four types of enzymatically converted DFAs, differentiated by the linkage between the fructose monomers: DFA I, α-D-fructofuranose-β-D-fructofuranose-2′,1:2,1′-dianhydride; III, α-D-fructofuranose-β-D-fructofuranose-2′,1:2,3′-dianhydride; IV, β-D-fructofuranose-β-D-fructofuranose-2′,6:2,6′-dianhydride, and V, α-D-fructofuranose-β-D-fructofuranose-2′,6:2,1′-dianhydride. DFA I, III, and V are converted from inulin (β-2,1 fructan) by the exo-acting transfructosylation of inulin fructotransferase (IFTase, EC 4.2.2.18), and DFA IV is converted from levan (β-2,6 fructan) by levan fructotransferase (LFTase, EC 4.2.2.16)^4^. Of these DFAs, DFA III is the most studied isomer and has been commercialised as the functional food “Twintose” in Japan. Similar to DFA III, DFA IV is regarded as a potential functional sweetener owing to its properties; it is colourless, odourless, has no cooling effect, and does not caramelise^1^.

The conventional processes for DFA IV production from sucrose involve levan preparation from sucrose by levansucrase (LSRase, EC 2.4.1.10), and DFA IV conversion from levan by LFTase. As the yield of LFTase increases with an increase in the degree of polymerisation (DP) of levan, bacterial levan that has a very high DP compared to plant levan^5,6^ has been favoured for the production of DFA IV. Therefore, microbial fermentation using strains expressing LSRase from *Bacillus subtilis* (Natural Polymers Inc., GA, USA), *Streptococcus salivarius* (Advance Co., Ltd., Tokyo, Japan), and *Zymomonas mobilis* (Real Biotech Co., Ltd., Chungnam, Korea)^7^ is predominantly used for the production of levan. The prepared levan is then reacted with LFTase obtained from microorganisms such as *Arthrobacter ureafaciens*, *A. nicotinovorans*, *A. oxydans*, and *Microbacterium* sp^1^.

To establish an efficient production system, Takesue *et al*. reported a single culture DFA IV production system from sucrose using a recombinant *B. subtilis* 168 strain expressing LFTase from *A. nicotinovorans*, and the strain successfully produced 43.5 g/L of DFA IV from an input of 250 g/L sucrose^8^. In addition, Kikuchi *et al*. developed a one-pot conversion of DFA IV from sucrose using two steps, comprising levan synthesis from sucrose by *Serratia levanicum* and crude LFTase from *A. nicotinovorans*, and achieved 6.8 kg of crude DFA IV from 30 kg of sucrose, similar to the highest conversion yield (45.3%) on the commercial scale^9^.

Baker’s yeast, *Saccharomyces cerevisiae*, is one of the most familiar workhorses for the production of various fermented foods in addition to its various biotechnological applications. This generally regarded as safe (GRAS) species has been extensively employed for recombinant protein production owing to the feasibility of secretion and post-translational modification. Furthermore, given the indigestible property of DFAs, this species has been used in the purification procedure of DFAs to remove residual monosaccharides^10,11^. To date, despite the favourable characteristics of *S. cerevisiae* as a DFA producer, there have been no reports on the application of *S. cerevisiae* for DFA IV conversion.

Herein, recombinant *S. cerevisiae* strains secreting LSRase from *B. subtilis* (BsSacB) and LFTase from *A. ureafaciens* (AuLftA) were constructed, respectively, by employing target protein-specific translational fusion partners (TFP) selected from a yeast genome-wide secretion leader library^12^. DFA IV was directly converted from sucrose by co-fermentation of two recombinant *S. cerevisiae* strains in one-pot with the highest titre, yield, and productivity ever reported.

## Results

### Construction of two recombinant yeast strains secreting BsSacB and AuLFT using the TFP technique

**Figure 1:**
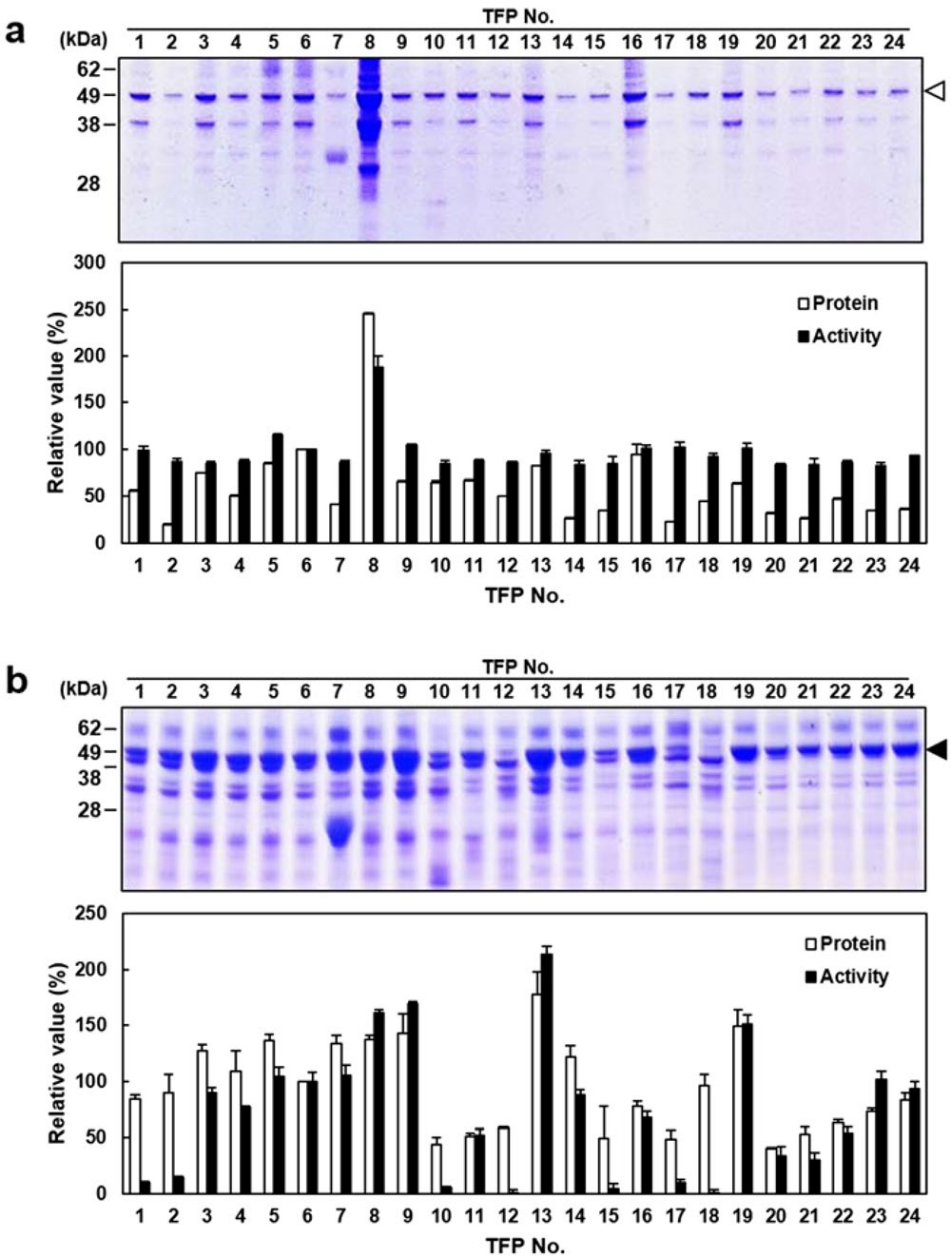
Selection of the optimal TFP for secretory production of BsSacB (**a**) and AuLftA (**b**). The amount of secreted protein was analysed by SDS-PAGE, and the relative amount of protein and activity was compared against mating factor alpha (TFP6).

Although BsSacB and AuLftA contain native signal peptides, the mature part of these enzymes was expressed under the control of *GAL10* promoter on a *URA3* selectable multi-copy vector by using 24 types of pre-selected TFPs (Supplementary Table S1), which are frequently screened for hypersecretion of recombinant proteins^13^. To qualify the TFPs, the mating factor alpha (MFα) pre-pro peptide of *S. cerevisiae* was contained as TFP6. The TFP vectors were designed to include the Kex2p processing site at the junction between the TFP and target proteins to secrete correctly processed proteins. Recombinant strains were directly constructed by co-transformation of linearised TFP vectors and the target genes flanked by the linker and terminator sequence. After cultivation of the transformants, the culture supernatants were analysed by SDS-PAGE and an activity assay to compare the secretion levels of BsSacB and AuLftA by the specific TFPs. As shown in Fig. 1, each recombinant protein band was detected with the corresponding molecular weight (BsSacB, 50.8 kDa; AuLftA, 54.6 kDa) indicating that precise Kex2p processing was performed as designed. TFP8 containing 64 amino acids (aa) from the N-terminus of the cell wall-associated mannoprotein SRL1 gave outstanding performance for the secretory production of BsSacB, and showed over 2-fold higher sucrose hydrolysis activity than that of TFP6 (MFα) (Figs 1a and S2). Therefore, the 2805gs/YGaTFP8-BsSacB was selected for the production of levan from sucrose.

Fot AuLftA, a high amount of AuLftA was produced in most of the TFPs (Fig. 1b). Therefore, the optimal TFP was screened through the relative DFA IV-forming activity from levan. As TFP13 containing 174 aa from the N-terminus of O-mannosylated heat shock protein HSP150 showed approximately 1.8-fold higher activity against TFP6 (MFα) (Figs 1b and S3), the strain harbouring YGaTFP13-AuLftA was selected for the production of DFA IV.

### Mass production of recombinant BsSacB and AuLftA by fed-batch fermentation

**Figure 2:**
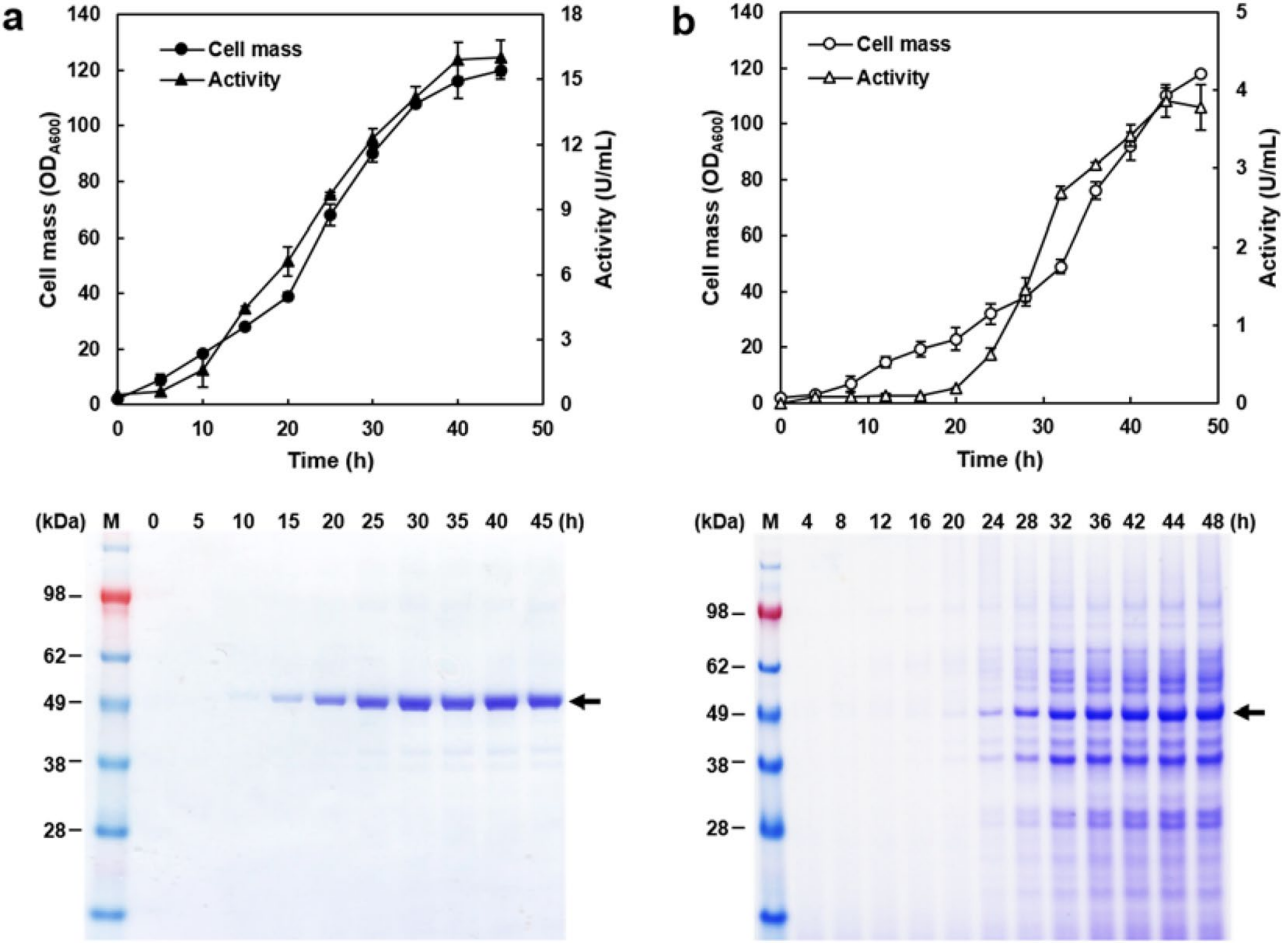
Mass production of recombinant AuLftA and BsSacB. In a 5-L jar fermenter, each recombinant yeast strain produced AuLftA (**a**) and BsSacB (**b**) into the culture media. Each cell mass was represented as circles, and the culture supernatant activity was indicated as triangles.

To guarantee high and constitutive expression of the *GAL10* promoter without galactose induction, and to inhibit sucrose hydrolysis by invertase, a *S. cerevisiae* strain disrupted in *GAL80* and *SUC2* (2805gs)^12^ was used for the mass production of recombinant BsSacB and AuLftA using a 5 L scale jar fermenter. As presented in Fig. 2a, the activity curve of AuLftA corresponded with the cell growth curve. The total amount of secreted proteins was calculated at approximately 600 mg/L and the maximum activity of the culture supernatant reached up to 16.0 U/mL. For BsSacB, the increase in activity was slowed during the exponential phase and 3.8 U/mL of glucose-liberating activity was detected after 48 h fermentation (Fig. 2b). The culture supernatants were analysed by SDS-PAGE without concentration. Contrary to AuLftA, which appeared as a distinct protein band, undesirable background protein bands were observed for BsSacB during fermentation at a high cell density. These background protein bands may have resulted from the degradation of BsSacB during fermentation, and the slight decrease in rate of increase of BsSacB activity can thus be explained by the degradation of BsSacB after the mid-log phase. Our results are supported by the frequent observation that the undesirable degradation of target proteins during secretory production in yeast species, with vacuole proteases regarded as the main cause of this^14,15^. Thus, host strain engineering or optimisation of fermentation conditions could increase the production of intact BsSacB. Scotti *et al*. tried to express BsSacB in *S. cerevisiae* using various signal peptides, but found that BsSacB was accumulated inside the cell in its precursor form^16^. This is the first successful secretory production of recombinant LFTase and BsSacB in a eukaryotic host cell. Both enzymes were tagged with hexa-histidine at carboxy terminus for the simple purification. Specific activity of a purified AuLftA by affinity chromatography was approximately 914.7 U/mg. But, unfortunately, BsSacB could not be purified by the same chromatography. We presumed that the C-terminal tag of BaSacB was not structurally exposed or not functioning by a possible proteolysis in yeast.

### Optimal sucrose concentration for microbial production of levan

**Figure 3:**
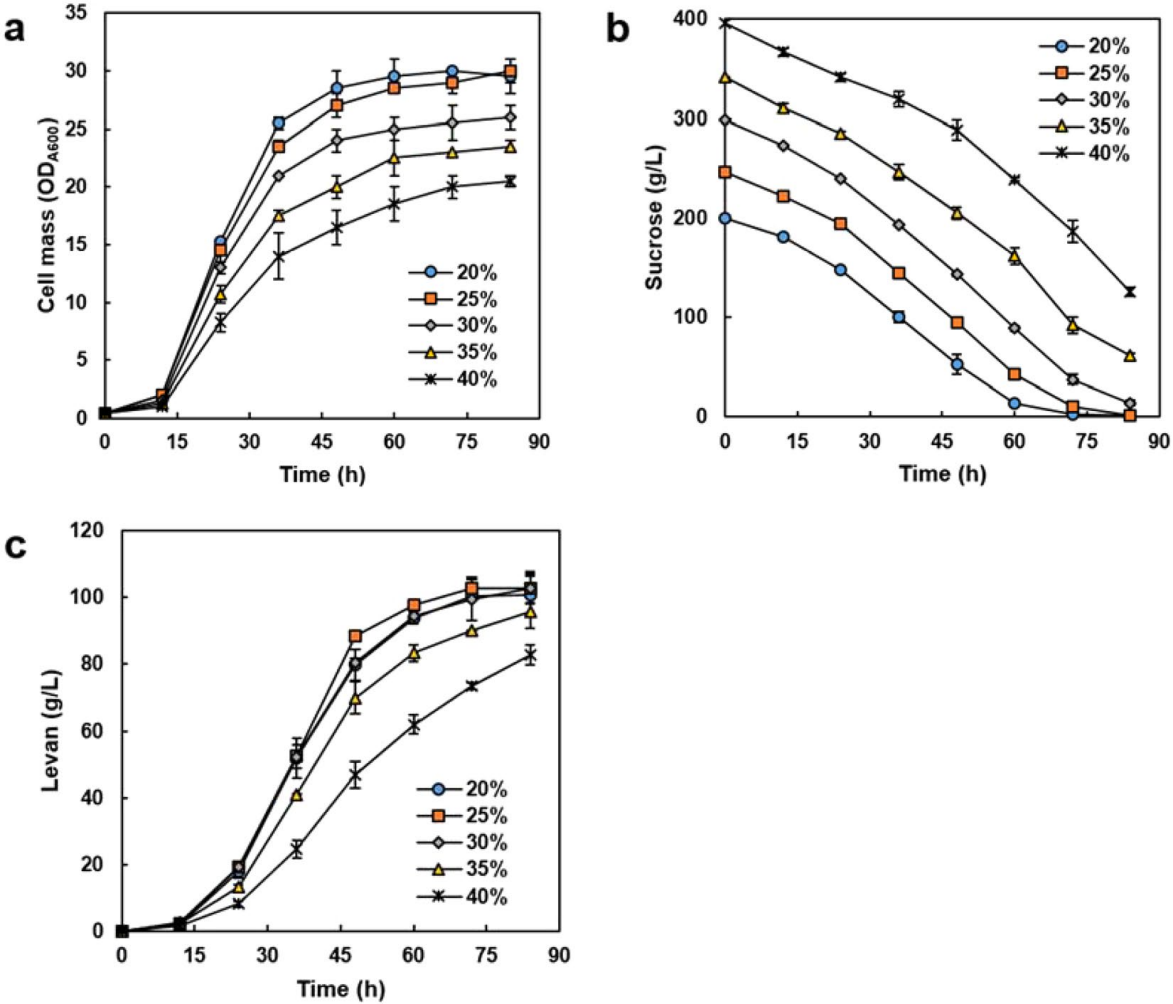
Optimal sucrose concentration for the direct production of levan by the recombinant yeast Y2805gs/YGaTFP8-BsSacB-6H. During incubation, cell growth dependent levan synthesis was observed. Cell growth, sucrose utilisation, and levan synthesis curve are presented in (**a–c**), respectively.

In YPS medium containing sucrose as the sole carbon source, invertase mutant *S. cerevisiae* strains expressing BsSacB can grow and synthesize levan, simultaneously, using glucose and fructose liberated from sucrose by the secreted BsSacB. Cell mass-dependent levan production was analysed at various concentrations of sucrose. Cell proliferation was inhibited at sucrose concentrations above 30% owing to osmotic stress (Fig. 3a). Although the utilisation rates of sucrose were similar regardless of sucrose concentration (Fig. 3b), the maximum titre was 102.9 g/L from 25–30% sucrose (Fig. 3c). Subsequently, the conversion yields decreased rapidly with an increase in sucrose concentration (Table 1). Similar results were demonstrated in the production of levan using native BsSacB^17^. The decrease in conversion yield at a high sucrose concentration implies that the sucrose-hydrolysing activity of levansucrase surpasses the levan-forming activity at high substrate concentration. As the molecular weight of polysaccharides is closely related to their solubility, 102.9 g/L would be the maximum solubility of levan produced by recombinant BsSacB. By gel permeation chromatography, the molecular weight of produced levan was estimated as 1.4 × 10^5^ Da. From 20% sucrose, 98.2 g/L of levan was synthesized. Considering the titre and conversion yield, a concentration of 25% sucrose was used for further experiments.

**Table 1:** Production of microbial levan from various concentrations of sucrose.

Initial sucrose (%)	Produced levan (g/L)	Conversion yield^a^ (%)	Time (h)	Productivity (g/L/h)
20	98.2 ± 2.2	98.2	84	1.17
25	102.9 ± 4.3	82.3	84	1.23
30	102.9 ± 4.8	68.6	84	1.23
35	95.7 ± 4.8	54.7	84	1.14
40	82.8 ± 2.9	41.4	84	0.99

^a^Conversion yield was calculated as produced levan against fructose based on initial sucrose.

### Mixed culture for direct production of DFA IV from sucrose

**Figure 4:**
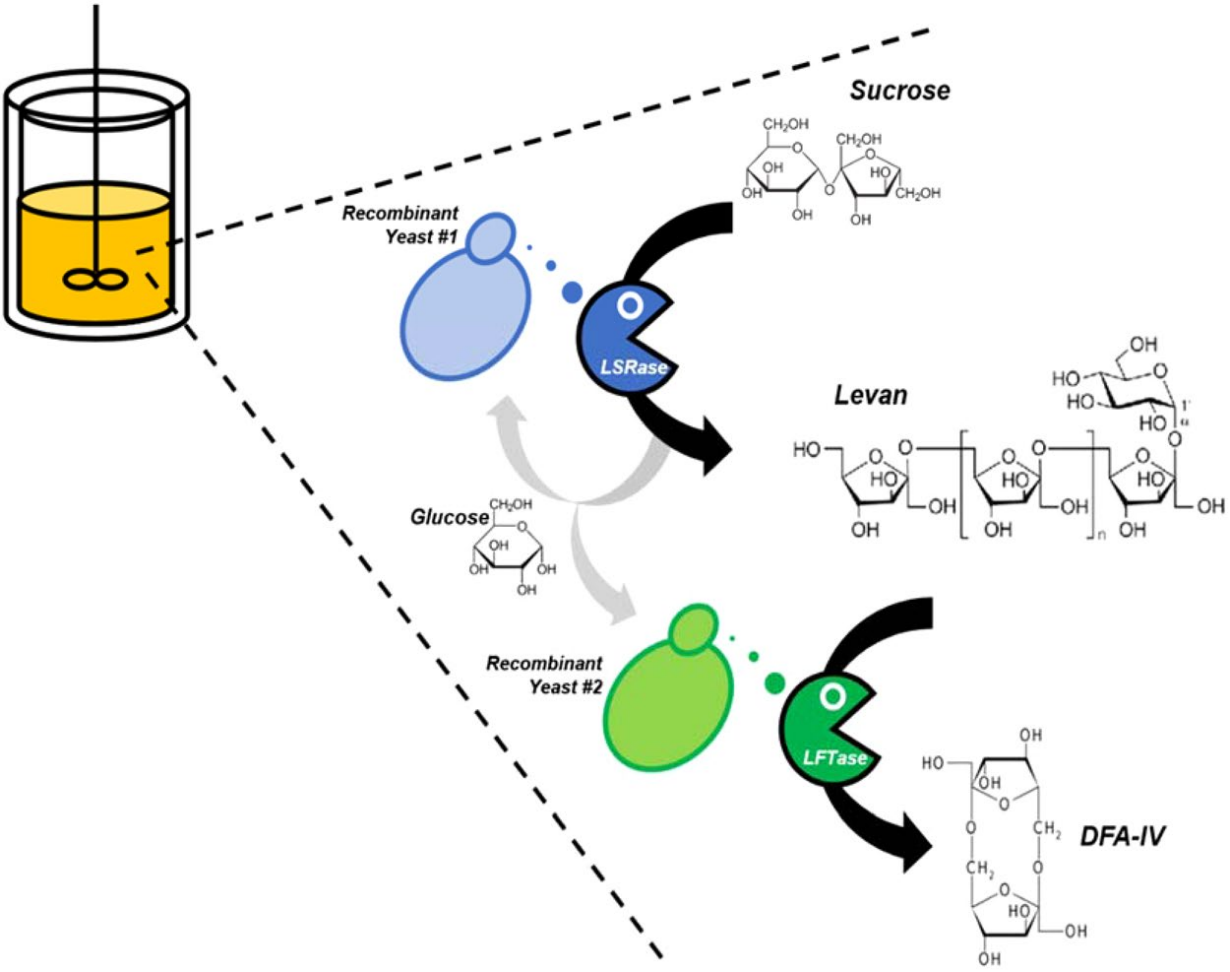
Scheme for the direct production of DFA IV by recombinant yeasts. In the fermenter, levan is synthesized from sucrose, and is converted to DFA IV, simultaneously, by the secreted LSRase and LFTase from two respective recombinant yeasts. Produced monosaccharides are consumed as carbon sources by yeast cells.

In the direct production system of DFA IV from sucrose by using a mixed culture of two recombinant yeast strains secreting BsSacB and AuLFT into the culture broth, DFA IV is converted from sucrose by the sequential reaction of BsSacB and AuLftA via levan (schemed on Fig. 4). Therefore, the amount of BsSacB and AuLftA should be optimised according to the specific activity of each enzyme. In the case of an enzymatic conversion system, the ratio could be easily determined by a kinetic study of the enzyme. However, in a microbial production system, this is unavailable because microbial fermentation is a complex system composed of various physicochemical factors. Therefore, we examined the optimal cell ratio for direct DFA IV production from 20% sucrose. The cell ratio was varied from 3:7 to 7:3 for each yeast strain (BsSacB:AuLftA), and the initial inoculum was adjusted to an optical density at 600 nm (OD_600_) = 1. As expected, the cell mass, sucrose  utilisation, and accumulated levan decreased with a decrease in the proportion of BsSacB, because cell growth is closely related to the activity of BsSacB (Table 2). With an increase in the proportion of AuLftA, the accumulated levan was rapidly decreased. In BsSacB-dominant conditions (B7:A3, B6:A4), sucrose was efficiently converted to levan by BsSacB, but large amounts of levan accumulated and DFA IV conversion was delayed due to the shortage of AuLftA. In AuLftA-dominant conditions (B4:A6, B3:A7), levan production was limited due the shortage of BsSacB; consequently, DFA IV production was decreased. Based on the titre of DFA IV, the optimal balance of the two recombinant yeast strains was 5:5.

**Table 2:** Cell ratio for the direct production of DFA IV.

Ratio (BsSacB:AuLftA)	Maximum cell mass (OD_A600_)	Unreacted sucrose (g/L)	Remained levan (g/L)	Produced DFA IV (g/L)	Conversion yield^a^ (%)	Time (h)	Productivity (g/L/h)
B7:A3	33.5 ± 1.5	n/d	63.8 ± 1.5	43.2 ± 0.8	43.2	84	0.51
B6:A4	31.5 ± 0.5	n/d	52.1 ± 0.9	50.4 ± 0.6	50.4	84	0.60
B5:A5	29.0 ± 0.5	9.5 ± 1.5	35.7 ± 1.6	56.8 ± 0.5	56.8	84	0.68
B4:A6	28.0 ± 1.0	50.2 ± 2.4	24.3 ± 1.7	49.4 ± 1.2	49.4	84	0.59
B3:A7	27.5 ± 1.5	89.7 ± 1.4	11.8 ± 0.1	39.0 ± 0.4	39.0	84	0.46

n/d, not detected.

^a^Conversion yield was calculated as produced DFA IV against initial fructose.

### Scale-up production of DFA IV in fermenter

**Figure 5:**
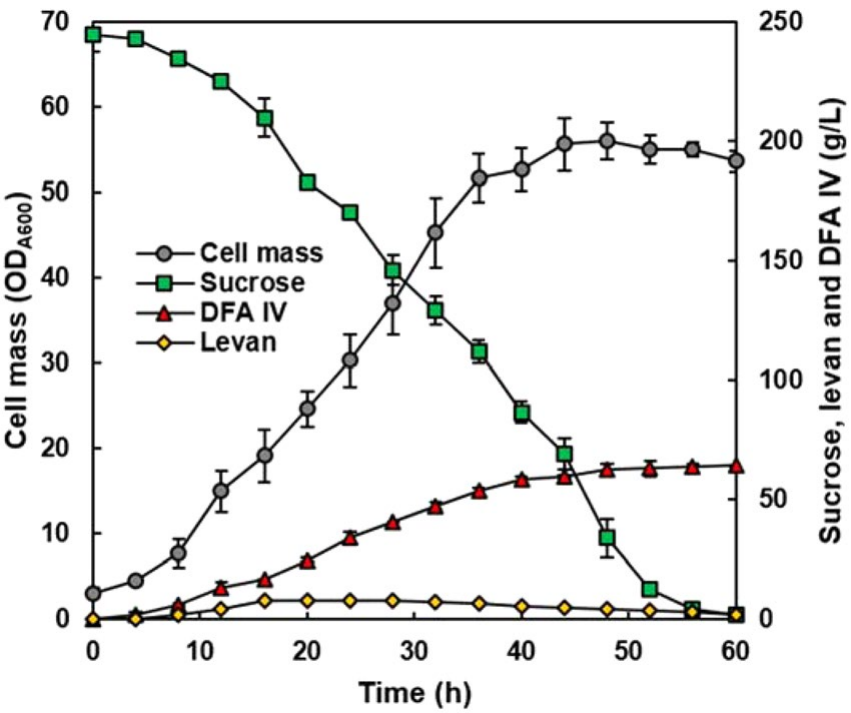
Direct production of DFA IV in a 5-L scale jar fermenter. Cell mass was analysed by optical density at 600 nm, and the amounts of sucrose, levan, and DFA IV were analysed using HPLC.

Direct production of DFA IV was demonstrated in a 5 L fermenter. As shown in Fig. 5, cell proliferation and DFA IV production were conducted simultaneously. From 244.7 g/L of sucrose, 64.3 g/L of DFA IV was converted after 60 h of culture. The cell mass reached approximately OD_A600_ = 56, which was higher than that in flask culture. The higher cell mass guarantees a larger quantity of recombinant enzymes, ensuring a high conversion titre of DFA IV. As shown in Table 3, the conversion yield was calculated to be 52.6%, and the productivity was 0.60 g/L/h.

**Table 3:** Comparison of DFA IV production using a one-pot system.

Method	Supplied sucrose (g/L)	Produced DFA IV (g/L)	Conversion yield^a^ (%)	Time^b^ (h)	Productivity (g/L/h)	Working volume (L)	Reference
Microbial	250	43.5	34.8	106	0.41	0.06	Takesue *et al*.^8^
Combined^c^	200	45.3	45.3	226	0.20	150	Kikuchi *et al*.^9^
Microbial	244.7	64.3	52.6	108	0.60	2	This study

^a^Conversion yield was calculated as produced DFA IV against initial fructose.

^b^Time was calculated as summation of the seed and main culture; the purification step was not considered.

^b^Combined method is a combination of microbial levan synthesis and enzymatic DFA IV conversion. Time for LFTase production was not included.

## Discussion

Previously, we developed a translational fusion partner (TFP) screening system for the secretory expression of recalcitrant proteins in *S. cerevisiae*^12^. We have subsequently succeeded in the secretory expression of various proteins, including industrial enzymes, by using this system^11–13^. In this study, we employed this system for the secretory production of bioactive BsSacB and AuLftA and TFP8 and TFP13 were selected as the optimal TFPs, respectively. TFP8 is the pre-secretion signal of SRL1 that is a putative GPI-anchored mannoprotein associated with cell wall stability^18,19^. TFP13 is the pre-pro-secretion signal of HSP150. HSP150 is a secretory glycoprotein induced by thermal stress^20^. Owing to its secretory properties, the N-terminal part of HSP150 was employed for the secretory production of rodent α-2,3-sialtransferase and *E. coli*-derived β-lactamase in yeast^21,22^. The secretion-enhancing mechanism of TFP remains to be determined, but is thought to assist in proper folding of the target protein in the endoplasmic reticulum and protein trafficking in the secretion pathway.

Recently, we produced recombinant LSRase from *Rahnella aquatilis* (RaLsrA) that has been rarely secreted in eukaryote hosts, and 15.1 U/mL of bioactive enzyme was successfully produced in fed-batch fermentation after application of the TFP technique^23^. At first, RaLsrA was employed for direct production of DFA IV from sucrose instead of BsSacB because RaLsrA has shown higher activity than BsSacB; however, contrary to expectations, the titre of DFA IV produced using RaLsrA was lower than that produced using BsSacB. Levansucrase mediates two reactions: hydrolysis and transfructosylation, and each reaction is influenced by reaction conditions, including pH, temperature, and sucrose concentration^24^. Therefore, a higher yield of DFA IV production by BsSacB/AuLftA than RaLsrA/AuLftA revealed that the transfructosylation activity of BsSacB may be higher than that of RaLsrA in the microbial fermentation conditions (pH 5.5, 30 °C with vigorous agitation).

To date, as described in the introduction, there are few reports on the direct production of DFA IV from sucrose in a single culture system. Takesue *et al*.^8^ reported the first success in the direct production of DFA IV from sucrose by using an engineered *B. subtilis* secreting an LFTase from *A. nicotinovorans*. By sucrose fed-batch fermentation, they produced 43.5 g/L of DFA IV from 250 g/L of sucrose with a 34.8% conversion yield. They suggested the degradation of produced levan by the SacC and LevU of *B. subtilis* as a reason for the low conversion yield of DFA IV. In our results, a higher DFA IV titre (64.3 g/L) and yield (52.6%) may be partially related to the used host, *S. cerevisiae*, which has no degradation enzymes for levan and DFA IV. In addition, constitutive hypersecretion of BsSacB and AuLftA facilitated by the TFPs enhanced the synthesis of levan and DFA IV during fermentation. Kikuchi *et al*.^9^ successfully produced DFA IV directly from sucrose with a yield of 45.3% in a one-pot system using a combination of microbial fermentation and enzymatic conversion. First, microbial fermentation was performed to produce levan from sucrose using *Serratia levanicum* NN; next, recombinant LFTase was added to the fermentation broth to convert levan to DFA IV. Despite the high conversion yield of DFA IV, they used a recombinant LFTase extracted from *E. coli* by lysozyme treatment and sonication, which would be challenging for large-scale industrial application. The secretory expression of LFTase in our system greatly simplified the production process and further improved the yield of DFA IV directly from sucrose.

The mixed culture of two recombinant yeasts secreting different enzymes was demonstrated to be effective in performing a sequential enzyme reaction to generate DFA IV from sucrose. However, we did not compare the effectiveness of the expression of the two proteins, BsSacB and AuLftA in a single yeast and in two yeasts, but maintaining different plasmids in a single cell is unfavourable for cell growth and poses difficulties in adjusting the relative expression of each protein. Simple control of the seeding ratio of the two yeasts allows for easy optimisation of the sequential enzyme reaction to convert sucrose to DFA IV. In general, due to the instability of episomal plasmids, the integrated expression of recombinant proteins is more favoured in industrial strains; however, our system showed a steady increase in DFA IV titre during prolonged fermentation (Fig. 5), implying that the cell ratio and expression plasmid were stably maintained. When the purified AuLftA was incubated in 4% levan solution, the DFA IV conversion yield increased to 73.5% (Supplementary Fig. S1), reaching the maximum theoretical value^25^. Consequently, there is additional room for further improvement of the DFA IV titre by optimizing the fermentation process. Considering the industrial production of DFA IV, our system can provide an economic and stable process as summarised in Table 3.

With the increasing demand for functional sweeteners, establishment of a straightforward and cost-effective production system for DFA IV is required. Herein, we have presented the direct production of DFA IV from sucrose by the co-culture of two recombinant yeasts, with the highest titre, yield, and productivity that have so far been reported.

## Methods

### Strains, chemicals, and media

*E. coli* DH5α [F^−^ *lac*ZΔM15 *hsd*R17(r- m-) *gyr*A36] was used for the recombinant DNA techniques. The parent strain, *Saccharomyces cerevisiae* (Y2805gs, *Mat α pep4::HIS3, prb1, can1, his3-200, ura3-52, gal80::Tc190, suc2::Tc190*) was used as the gene expression host to avoid sucrose hydrolysis^12^. DNA polymerase and restriction enzymes were purchased from New England Biolabs (Ipswich, MA, USA). DNA purification was conducted using the Wizard® SV Gel and PCR Clean-Up Systems (Promega, Madison, WI, USA). Levan and DFA IV standards were purchased from Realbiotech Co., Ltd. (Gongju, Korea). Various molecular weight dextran standards (12,000 to 670,000 Da) were purchased from Sigma-Aldrich (St. Louis, MO, USA). Yeast cells were cultured in YPD (1% yeast extract, 2% Bacto peptone, and 2% glucose). Yeast transformants were screened on a synthetic defined medium lacking uracil (SD-Ura; 0.67% yeast nitrogen base without amino acids, 0.077% Ura dropout supplement, 2% Glucose, and 2% Bacto agar).

### Construction of recombinant yeast strains secreting BsSacB and AuLftA by employing the TFP technique

The mature part of target genes was synthesized with the homologous recombination region and six-histidine tag at the 5′ and 3′ end, respectively, by Bioneer Corp. (Daejeon, Korea) based on the NCBI database (BsSacB, ARE67151.1; AuLftA, AAF73829.1). Each gene was amplified by PCR (forward primer: 5′- GGCCGCCTCGGCCTCTGCTGGCCTCGCCTTAGATAAAAGA-3′, reverse primer: 5′-GTCATTATTAAATATATATATATATATATTGTCACTCCGTTCAAGTCGACTTAGTGATGGTGATGGTGATG-3′), and purified. The genes and linearised TFP vectors^13^ were introduced into the yeast host strains, respectively, by using the lithium acetate method^26^. Recombinant vectors were constructed by *in vivo* recombination. To compare the secretion capacity of target proteins, selected transformants from the SD-Ura plate were cultured in 3 mL YPD broth at 30 °C, 200 rpm, for 40 h. Then, 0.6 mL of the culture supernatant was mixed with 0.4 mL of ice-cold acetone, and incubated at −20 °C for 2 h. The protein pellet was collected by centrifugation at 10,000 × *g* for 15 min, and lyophilised. Protein samples were dissolved in 10 μL of 1× Laemmli sample buffer (Bio-Rad, Hercules, CA, USA), and analysed on 4%–12% gradient Tris-glycine gels (ThermoFisher Scientific, Waltham, MA, USA). The relative amount of secreted proteins by TFPs was analysed by using ImageJ software^27^.

To determine LSRase activity, 100 μL of culture supernatant was added to 900 μL of 1% sucrose solution, and the mixture was reacted at 30 °C for 1 h. The reaction was then stopped by heat shock at 95 °C for 15 min, and the produced glucose was analysed by HPLC. LFTase activity was compared with respect to the DFA IV-forming capability. For this, 100 μL of the culture supernatant was mixed with 900 μL of 1% levan solution, and incubated at 30 °C for 10 min. The reaction was stopped as described above, and the produced DFA IV was analysed by HPLC.

### Fed-batch fermentation of recombinant BsSacB and AuLftA

The mass production of BsSacB and AuLftA from recombinant yeast strains was performed by fed-batch fermentation, respectively. As the seed culture, the recombinant yeast cells were incubated in a 250-mL flask containing 50 mL SD-Ura broth medium at 30 °C, 180 rpm, for 24 h, and the culture was transferred to a 1-L flask containing 200  mL YPD broth and was incubated for 24 h in the same conditions. A total of 0.25 L seed culture was inoculated into 1.75 L main medium (3% yeast extract, 1.5% peptone, and 2% glucose) in a 5-L jar fermenter (CNS Inc., Daejeon, Korea). Fermentation conditions were maintained at 30 °C, 900 rpm, and 1 vvm. The pH was adjusted to 5.5 using ammonium hydroxide. During fermentation, feeding medium (5% yeast extract and 30% glucose) was added according to the glucose concentration. Culture samples were collected every 4 h for analysis of cell mass (OD_A600_) and enzyme activity.

### Determination of optimal conditions for microbial DFA IV production

To investigate the optimal sucrose concentration for microbial levan production, Y2805gs/YGaTFP8-BsSacB-6H was cultivated in 50 mL YPS medium (1% yeast extract, 2% peptone, 2%–40% sucrose) at 30 °C, 180 rpm, for 84 h. During incubation, samples were collected every 12 h for determination of cell mass and quantitative HPLC analysis. The molecular weight of the produced levan was determined by using gel permeation chromatography and HPLC. To optimise the cell ratio for direct production of DFA IV, two recombinant yeast cells were incubated in 50 mL of YPS medium (1% yeast extract, 2% peptone, and 20% sucrose) at various ratios of inoculum volumes, ranging from 3:7 to 7:3 (BsSacB:AuLftA secreting yeasts). The initial OD_A600_ was adjusted to 1. After 84 h incubation, the cell mass was analysed by OD_A600_, and quantitative analysis of sucrose, levan, and DFA IV was performed by HPLC.

### Direct production of DFA IV from sucrose in the fermenter

Direct production of DFA IV was demonstrated using a 5-L scale jar fermenter (2 L working volume). Each recombinant yeast strain was cultivated in 50 mL SD-Ura broth at 30 °C, 180 rpm, for 24 h, and then transferred into 200 mL YPD broth culture and incubated in the same conditions. In total, 500 mL of seed culture was inoculated in 1.5 L of main medium (1% yeast extract, 2% peptone, and 25% sucrose). The fermentation conditions were maintained at pH 5.5, 30 °C, 900 rpm, and 1 vvm. During fermentation, the culture medium was collected every 4 h, for analysis of cell mass and products. To investigate the amount of sucrose and DFA IV, the medium was centrifuged at 13,000 × *g* for 3 min, and the supernatant was analysed by HPLC.

### HPLC analysis

The HPLC-RID system (Agilent 1100 series, Agilent Technologies, Santa Clara, CA, USA) was employed for quantitative analysis of levan, sucrose, DFA IV, and other saccharides. Sucrose and DFA IV were analysed by using YMC-PACK ODS AQ (YMC Co., Ltd., Kyoto, Japan). To detect levan and other saccharides, Sugar KS-802 (Showa Denko K. K., Tokyo, Japan) was used. An estimation of the molecular weight of produced levan was performed using Sugar KS-806 (Showa Denko K. K.). HPLC-grade water was used as the mobile phase with a 0.5 mL/min flow rate for saccharide analysis and a 1.0 mL/min flow rate for molecular weight analysis.

## Supplementary information

Supplementary Information
