# Supplementary material for: Direct Production of Difructose Anhydride IV from Sucrose by Co-fermentation of Recombinant Yeasts
Source: Sci Rep. 2019 Nov 4;9:15980. doi: 10.1038/s41598-019-52373-5 (PMC6828762; doi:10.1038/s41598-019-52373-5)

**Supplementary Information**

**Direct Production of Difructose Anhydride IV from Sucrose by Co-fermentation of Recombinant Yeasts**

Hyunjun Ko^1,2^, Jung-Hoon Bae^1^, Bong Hyun Sung^1,2^, Mi-Jin Kim^1^, Soon-Ho Park^1^ & Jung-Hoon Sohn^1,2,*^

**Supplementary Table S1.** Brief information of TFPs used in this study

**Supplementary Fig. S1.** Full-length SDS-PAGE gels of optimal TFP selection for secretory production of BsSacB (a) and AuLftA (b).

**Supplementary Fig. S2.** Comparison of secretion level and activity of BsSacB from three different colonies secreting BsSacB under the control of eight selected TFPs from Fig. 2(A)

**Supplementary Fig. S3.** Comparison of secretion level and activity of AuLftA of recombinant cells secreting AuLftA under the control of six selected TFPs from Fig. 2(B).

**Supplementary Fig. S4.** Enzymatic conversion of DFA IV from levan by the recombinant AuLftA produced in this study.

**Supplementary Table S1.** Brief information of TFPs used in this study.

| TFP No. | Gene name | Length^a^ | Characteristics^b^ |
| --- | --- | --- | --- |
| 1 | YAR066 | 118 | Pre-SS, N-Gly, SA-rich, GPI |
| 2 | YFR026c | 130 | Pre-SS, N-Gly, TMD |
| 3 | CIS3 | 117 | Pre-pro-SS, O-gly, PIR |
| 4 | DAN2 | 66 | Pre-SS, CWP |
| 5 | SCW4 | 97 | Pre-SS, CWP |
| 6 | MFα | 93 | Pre-pro-SS |
| 7 | YGR106C | 226 | Pre-SS, N-Gly, TMD |
| 8 | SRL1 | 64 | Pre-SS, N-Gly, O-Gly, SA-rich |
| 9 | SIM1-1 | 138 | Pre-SS, N-Gly, O-Gly, SA-rich, SUN family |
| 10 | OST3 | 199 | Pre-SS, O-Gly |
| 11 | Ynl190w | 77 | Pre-SS, N-Gly, internal repeats, CWP |
| 12 | EMP24 | 94 | Pre-SS, TMD |
| 13 | HSP150 | 174 | Pre-pro-SS |
| 14 | ECM33 | 68 | Pre-SS, GPI |
| 15 | ATG27 | 157 | Pre-SS, TMD |
| 16 | UTH1 | 98 | Pre-SS, SUN family, S-rich |
| 17 | SED1 | 195 | Pre-SS, GPI |
| 18 | BGL2 | 91 | Pre-SS |
| 19 | SCW4 | 124 | Pre-SS, CWP |
| 20 | CCW12 | 138 | Pre-SS, CWP |
| 21 | FIT3 | 176 | Pre-SS, GPI |
| 22 | YGP1 | 138 | Pre-SS, N-Gly, CWP |
| 23 | CCW14 | 115 | Pre-SS, CWP |
| 24 | SED1 | 170 | Pre-SS, GPI |

^a^Number of amino acids

^b^Pre-SS, pre secretion signal; Pre-pro-SS, pre-pro secretion signal; N-Gly, N-glycosylation potential; O-Gly, O-glycosylation potential; SA-rich, serine-alanine rich domain; S-rich, serine rich domain; GPI, glycosyl phosphatidyl inositol anchor protein; TMD, transmembrane domain; PIR, protein internal repeats; CWP, cell wall protein

**Supplementary Fig. S1.** Full-length SDS-PAGE gels of optimal TFP selection for secretory production of BsSacB (a) and AuLftA (b).


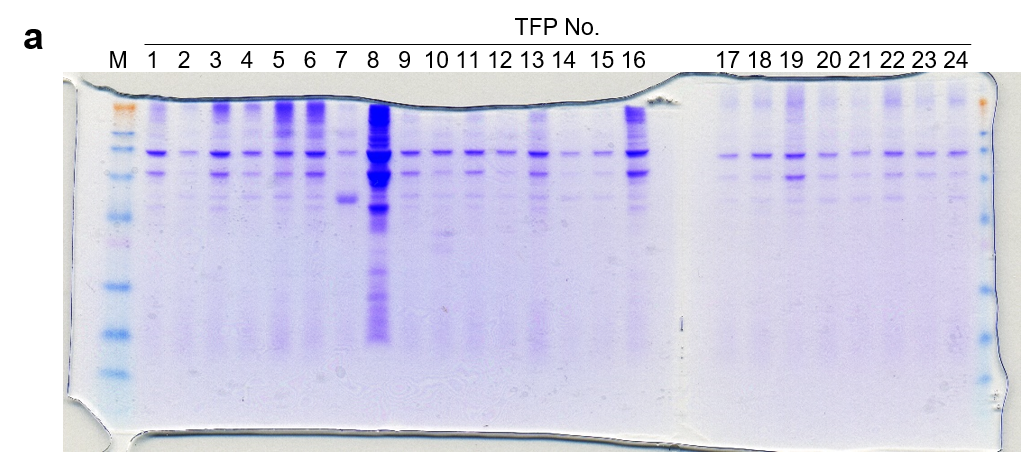

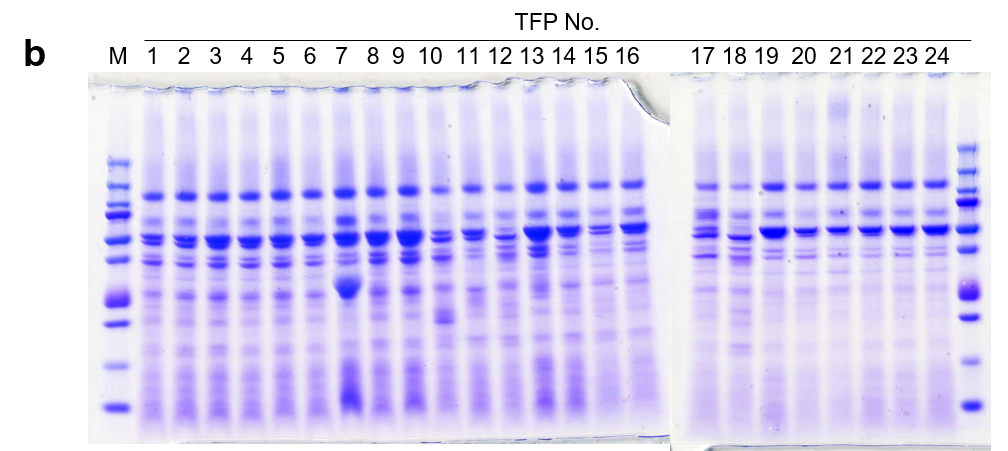


**Supplementary Fig. S2.** Comparison of secretion level and activity of BsSacB from three different colonies secreting BsSacB under the control of eight selected TFPs from Fig. 2(A).


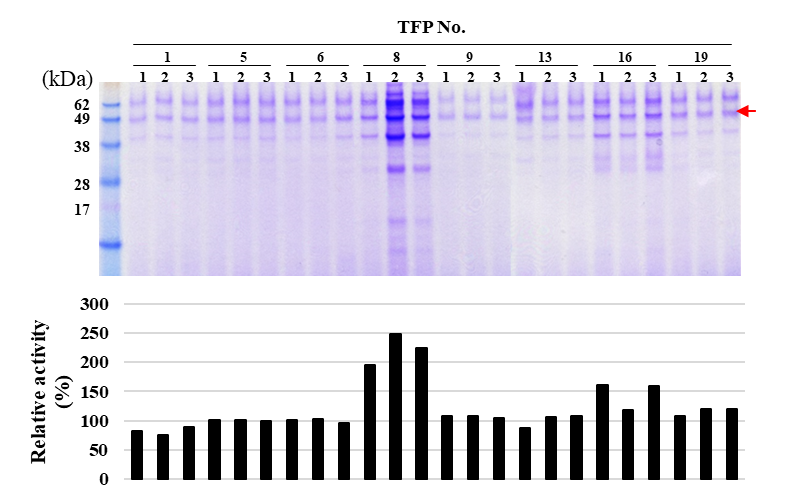


**Supplementary Fig. S3.** Comparison of secretion level and activity of AuLftA of recombinant cells secreting AuLftA under the control of six selected TFPs from Fig. 2(B).


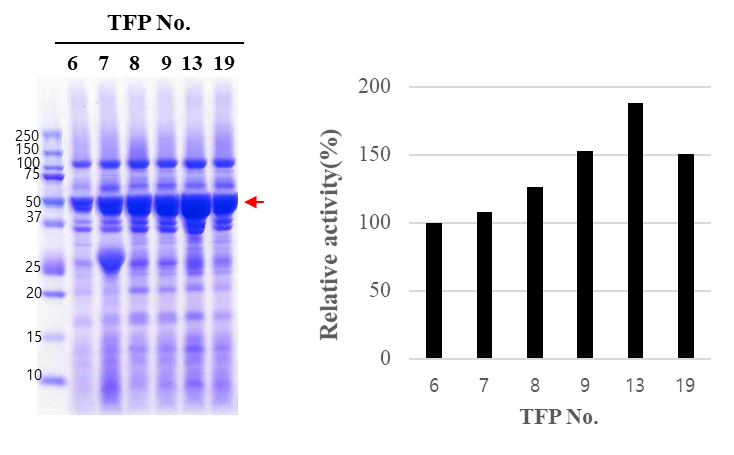


**Supplementary Fig. S4.** Enzymatic conversion of DFA IV from levan by the recombinant AuLftA produced in this study.


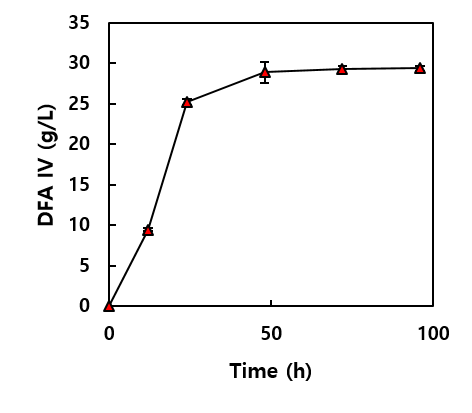

Supplement: Supplementary file 1 — Supplementary Information [file 41598_2019_52373_MOESM1_ESM.docx]
